# Zusammenhänge zwischen Empathie, therapeutischer Haltung und Wirkeffizienz

**DOI:** 10.1007/s00739-021-00726-z

**Published:** 2021-04-22

**Authors:** Dagmar Steinmair, Henriette Löffler-Stastka

**Affiliations:** 1 Abteilung für Psychoanalyse und Psychotherapie, Medizinische Universität Wien, Währinger Gürtel 18–20, 1090 Wien, Österreich; 2 Karl Landsteiner Privatuniversität für Gesundheitswissenschaften, Dr. Karl-Dorrek-Straße 30, 3500 Krems an der Donau, Österreich

**Keywords:** Verkörperte Empathie, Theorie des Geistes, Soziale Emotionen, Unbewusstes, Soziale Kognition, Embodied empathy, Theory of Mind, Social emotions, Unconscious, Social cognition

## Abstract

Empathisch sein heißt, fühlen und verstehen können, was andere fühlen. Vermuten zu können, was das Gegenüber fühlt, denkt und wünscht, beruht auf der Fähigkeit, eigene Gefühle und Gedanken als getrennt von jenen anderer wahrnehmen und regulieren zu können. Definierte Therapieerfolge mit adäquatem Aufwand erreichen zu können, verlangt ein Fokussieren auf Wesentliches und Wichtiges. Die Empathie ist im Bereich der Psychotherapie jener Faktor, für den für sich genommen die höchste Effektstärke nachgewiesen werden konnte. Empathietraining ermöglicht eine bessere soziale Performance. Im Falle von Defiziten in sozialer Kompetenz ist störungsunabhängig ein besonders hoher Leidensdruck nachweisbar.

## Abstract (en)

Empathy means feeling and understanding what others are feeling. Being able to imagine what the other person feels, thinks, and wishes is based on the ability to sense and regulate one’s own feelings and thoughts separately from those of others. Achieving desired therapy outcomes with adequate effort requires focussing on what is essential and important. In the field of psychotherapy, empathy is the one factor with the most significant effect size. Empathy training enables better social performance. In the case of social skills deficits, a particularly high level of distress is demonstrated, independent of the mental health condition.

## Einleitung

Empathie stellt die Grundlage für soziales und moralisches Verhalten dar – häufig wird sie als „einfühlendes Verstehen“ definiert und beinhaltet die Fähigkeit, die Perspektive und Erfahrungswelt anderer wahrnehmen zu können und sie von der eigenen zu unterscheiden [1].

Empathische Kommunikation beeinflusst Patientenzufriedenheit, Lebensqualität und Schmerzempfinden von Patienten positiv [2–9]. Bisherige Studien zur Kosten- und Wirkeffizienz von Empathie zeigten des Weiteren, dass Vorteile von Empathie evident sind, dass aber eine Erforschung weiterer Zusammenhänge empfehlenswert ist [6, 10]. Empathische Kommunikation reduziert Stress für Patienten und erhöht das Vertrauen in Behandler [11, 12]; Depressivität, Angst, das Risiko für Herzerkrankungen und Mortalität sinken [10–13]. Indirekte Kosten werden gesenkt durch bessere Diagnosen, höhere Therapie-Adhärenz, geringere Hospitalisierungsraten und -dauer [10, 12, 14]. Nicht zuletzt bedeutet ein empathischer Umgang auch für Behandler ein geringeres Burn-out-Risiko sowie eine höhere klinische und rechtliche Sicherheit [15–17]. Im Alltag kann ein Fokus auf empathische Kommunikation einen zeitlich geringgradigen Mehraufwand bedeuten [10]. In psychotherapeutischen Settings kommt ohne Empathie auf Therapeutenseite keine heilsame therapeutische Beziehung zustande, diese ist für einen Therapieerfolg notwendig, aber alleine nicht ausreichend [18]. Empathie wurde in der vergleichenden Betrachtung von allgemeinen und spezifischen Wirkfaktoren als der Faktor mit der größten Effektstärke für den Therapieerfolg identifiziert [18].

## Empathie und Mitgefühl

Eine Unterscheidung zwischen Mitgefühl und Empathie wird getroffen, um negative Auswirkungen von Empathie hervorzuheben. Empathisches Sich-Einfühlen und Nachempfinden kann dazu führen, dass es schwierig wird, eigene Wünsche und Bedürfnisse von jenen anderer abzugrenzen. Training und Supervision haben bei Psychotherapeuten eine positive Auswirkung auf Lebensqualität und Resilienz gezeigt [19].

Erfahrene Therapeuten zeigen eher ein „rational empathisches“ Profil

Eine Analyse der Ausprägung von klinischer Empathie bei Experten (*N* = 775) [20] erhob der Interpersonal Reactivity Index, welcher Perspektivenwechsel (PW), Fantasiefähigkeit (F), empathische Besorgnis (EB) und persönlichen Distress (PD) misst. Die beschriebenen Therapeutenprofile: 1: 23 % „insecure self-absorbed“ (unsicher/mit sich selbst beschäftigt: höchster PD, unterdurchschnittlich bei PW, F, EB); 26 % „empathic immersion“ (intuitiv, sich identifizierend: höchste EB und überdurchschnittlich bei PW, F); 38 % „average“ (durchschnittlich bei PW, F, EB und PD) und 13 % „rational empathic“ (intellektuell verstehend: höchster PW, durchschnittliche EB, geringe F und geringster PD) [20]. Eine multidimensionale Betrachtung empathischer Fähigkeiten zeigt, dass übermäßiges Nachfühlen und Sich-Identifizieren mit dem Patienten zusätzlich zum notwendigen PW dem Therapieerfolg abträglich ist. Damit übereinstimmend: Erfahrene Therapeuten zeigten eher ein „rational empathisches“ Profil.

Mit Empathie ist aus psychoanalytischer Sicht ein Set unbewusster und intuitiv ablaufender intra- und interpersonaler Vorgänge gemeint [21]. Philosophische Konzeptualisierungen von Empathie beinhalten neben einer kognitiven Komponente (z. B. Theory of Mind [ToM], inkl. PW) eine phänomenologische, erfahrungsbasierte Komponente (vgl. „embodied empathy“ – z. B. Betonung körperlicher Resonanz und Affektivität) [22–24]. Seelische Belastung, falsche Konsens-Effekte [25] und Missverständnisse führen zu egozentrischem PW. Ausgehend von eigenen Erfahrungswelten werden jene anderer unzureichend simuliert; dies kann zu emotionaler Überempfindlichkeit führen [24, 26, 27], welche in sozialen Interaktionen relevant wird.

Im Laufe des Medizinstudiums wurde für Studenten eine abnehmende Empathiefähigkeit in Selbsteinschätzungen dokumentiert [28, 29]. Stress führt zu Burn-out sowie „compassion fatigue“ in helfenden Berufen [30–32]. Ob Interventionen, um kommunikative Fähigkeiten und Empathie zu trainieren effektiv sind, wird kontrovers diskutiert – vor allem besteht noch kein Konsens darüber, welche Methodik hierfür am geeignetsten ist, auch zu Langzeiteffekten fehlen noch konsistente Resultate; der in einer Metaanalyse nachgewiesene signifikante Trainingseffekt war für personalisiertes und sehr spezifisches Feedback nachweisbar [27, 33]. Arbeiten zur „wahrgenommenen Verhaltenskontrolle“ gehen davon aus, dass die subjektive Überzeugung und die Erfahrung, Ressourcen zu haben, ein Verhalten auszuführen, dazu beitragen, das Verhalten auch zu realisieren [34]. Eine Erhebung von Fertigkeiten bei Medizinstudenten in Österreich zeigt Verbesserungspotenzial; etwa, dass nur die Hälfte der evaluierten Abteilungen den Studierenden einen konstanten Mentor zugeteilt hatte [35]. Unklarheiten bei Studierenden zu Konzept und Relevanz von Empathie zeigte die Metaanalyse von Costa-Drolon et al. [36]. Patientenrückmeldungen bzgl. der Empathie ihrer Behandler zeigen eine hohe Variabilität [37].

Psychoanalytische Behandlungsmethoden fokussieren auf Training von Containment, Fähigkeit zur Mentalisierung und Affektregulation unter Berücksichtigung von Übertragung und Gegenübertragungsprozessen [21]. Wohlbefinden und Therapieerfolge stehen in engem Zusammenhang mit zum Teil adaptiven Persönlichkeitseigenschaften wie Resilienz, Optimismus und durch Erfahrung gewonnene Weitsicht [38].

Das beobachtete Verhalten von Bezugspersonen hat Einfluss auf Distress-Symptome bei Kindern während medizinischer Eingriffe. In unter Zweijährigen wirkt sich fehlende mitfühlende Nähe während der Maßnahmen negativ aus, während bei älteren Kindern der Distress steigt, wenn negative und bedrohliche Aspekte der Situation ins Zentrum der Aufmerksamkeit gerückt werden [39].

„Weisheit“ beinhaltet, neben kognitiv-intellektuellen Aspekten, soziale Kompetenz, Emotionsregulation, Ambivalenztoleranz, Reflexionsfähigkeit und Entschlossenheit; sie zeigt sich in der Anwendung von Wissen in Übereinstimmung mit sozialen und persönlichen Interessen [38]. Eine Metaanalyse zeigt, dass prosoziales Verhalten und Emotionsregulation signifikant trainierbar sind [38].

Die willentliche Entscheidung, eine empathische Haltung einzunehmen, scheint nicht ausreichend, um empathisches Verhalten zu erreichen [33, 34, 40], allerdings wird für die kognitive Empathie eine bewusste Steuerbarkeit berichtet. Unbewusste zwischenmenschliche Prozesse sind jedoch unabhängig von Persönlichkeitsmerkmalen und kognitiver ToM. Dies widerspricht Studienergebnissen, welche Menschen mit bestimmten Persönlichkeitsmerkmalen den Willen zum empathischen Handeln, aber nicht die Empathiefähigkeit absprachen [41]. Soziale Emotionen sind jene, welche nicht durch individuelles Erleben evoziert werden, sondern durch die Zugehörigkeit zu einer Gruppe und zu assoziierten Ereignissen unter der Voraussetzung der Ausprägung einer sozialen Identität (z. B. Stolz, Freude, Schuld, Scham, Ingroup-Angst und Wut/Hass auf eine Outgroup [42]). Entsprechend basiert Training von prosozialem Verhalten auf Beobachtung und Imitation sozialer Emotionen; dies konnte bei schizophrenen Patienten für die kognitive als auch für die affektive ToM gezeigt werden [43]. Eine Verbesserung der „social cognition“ durch ein Training der Mentalisierungsfähigkeit bei Ärzten und Therapeuten einer psychiatrischen Abteilung konnte mittels videobasiertem Assessment nachgewiesen werden (Movie for the Assessment of Social Cognition) [27, 44].

## Kasuistik

Folgendes Fallbeispiel dient dazu, das Konzept der mentalisierten Affektivität und Defizite in dieser Fähigkeit zu beleuchten. Am Beispiel der Schilderung des Falls A. sollen Herausforderungen einer ambulanten Patientenbetreuung aufgezeigt werden. Frau A., Anfang dreißig, abwechselnd als paranoid, negativistisch und „Borderline“ beschrieben, war nach stationärer Behandlung nach einem Suizidversuch in Psychotherapie. Auffallend waren eine Selbstvernachlässigung und bösartige, aber amüsante Kommentare über andere sowie konfuse Assoziationen. Ein Misshandlungsgeschehen in Frau A.s Jugend war anamnestisch bekannt. Die Arbeit hatte sie verloren, die Umgebung und die Mitmenschen ließ sie durch ihr wegstoßend, zerstörerisches und zum Teil beschuldigendes Verhalten leiden. Im Zuge der Therapieplanung verhielt sie sich selbstdestruktiv – ausgelöst durch eine Unfähigkeit, Affekte zu regulieren. Zu Beginn der Therapie waren besonders die Fehlinterpretation des Verhaltens anderer (z. B. Unterstellung böser Absicht, leichte Kränkbarkeit) sowie der überwiegend negative Affekt (v. a. Wut) und eine desorganisierte Bindung mit Isolation auffallend. Sie vernachlässigte ihren Gesundheitszustand, heizte die Wohnung nicht und wurde wegen einer Pneumonie internistisch stationär behandelt. Während Frau A. eine Begegnung mit dem Konsiliarpsychiater schilderte, in welcher sie auf die Frage: „Denken Sie manchmal daran, sich selbst etwas anzutun?“, geantwortet hatte: „Nein, ich denke daran, das Spital zu klagen!“ nahm die Therapeutin eine Überflutung der eigenen Psyche mit Frau A.s Gefühlen wahr. Frau A. musste die Standardfrage als Affront interpretiert haben. Der Aufbau einer therapeutischen Beziehung gelang – wobei die Therapeutin Schwierigkeiten mit der eigenen Affektregulierung im Sinne einer Gegenübertragungsreaktion feststellte. Frau A.s teils mit Humor abgewehrte Aggression bot der Therapeutin eine Möglichkeit, Kontakt herzustellen. In der Therapie äußerte sie basierend auf der Beobachtung einer aus ihrer Sicht müde wirkenden Therapeutin: „Sehen Sie sich doch mal an! Haben Sie denn überhaupt keine Ahnung … Sie könnten sterben, wenn Sie so … mein Blick könnte sie töten“. Die Äußerung legt nahe, dass Frau A. die scheinbare „Müdigkeit“ der Therapeutin als auch für sie bedrohlich oder als Provokation empfand. Die Feststellung beendet sie mit der Empfehlung, die Therapeutin solle sich einen Kaffee holen. Gewissermaßen soll die Bedrohung der Therapeutin durch ihren Blick wiedergutgemacht werden. Die fürsorgliche aber triumphierende Äußerung kann als konstruktiver Umgang mit Aggression und Libido betrachtet werden. Das notwendige Containment destruktiver Impulse und Affekte muss immer wieder geleistet und in Worte gefasst werden.

## Diskussion und Ausblick

Interesse an der eigenen inneren Welt und jener des Patienten ist eine Voraussetzung für eine lebendige Erfahrung in der Therapie, welche sich vom theoretischen Verstehen einer Situation unterscheidet. Identifizierung, Modulierung und Äußerung von Affekten bedingen empathisches Verstehen und Verhalten. Therapeutisch wird an der Einstellung des Patienten zu seiner Affektivität gearbeitet. Im Falle einer projektiven Identifizierung, wie sie im oben beschriebenen Beispiel dargestellt ist, wird die Psyche des Therapeuten durch unerträgliche und widersprüchliche Affekte des Patienten überflutet, die durch die Patientin nur zum Teil wahrnehmbar und nicht integrierbar blieben (vgl. auch [45]). Ohne die Möglichkeit, Gedanken als Vorstellungen in einem Möglichkeitsraum zu repräsentieren, ist der Mensch zum (repetitiven) Agieren prädisponiert. Die Entfaltung des Selbst als auch der Aufbau einer Beziehung zu anderen hängen entscheidend davon ab, ob die eigene innere Welt als solche abgrenzbar, kohärent und beherrschbar ist oder ob sie mit jener des Gegenübers verschwimmt.

Identifizierung, Modulierung und Äußerung von Affekten bedingen empathisches Verstehen und Verhalten

Angehende Mediziner sehen empathische Fähigkeiten als wichtig an, allerdings variiert diese Haltung je nach gewählter fachärztlicher Spezialisierung [46]. Bei Medizinstudenten wurden Alter, Geschlecht, Land, Zweck und Art der Intervention/Messung und das Vorhandensein einer Trainingsmöglichkeit als Moderatoren der Empathie erhoben [47].

Perspektivenwechsel lassen sich auch in einem „virtual reality training“ signifikant verbessern – allerdings ist die erzielte Verbesserung nicht signifikant für Empathie [48]. Bei der Erstellung von E‑Learning-Fällen gilt es neben der Vermittlung von theoretischem Wissen (vgl. deklaratives Gedächtnis), auch die klinische Perspektive erfahrbar zu machen [49], wobei die Vermittlung von Skills (vgl. prozedurales Gedächtnis) und affektiver ToM via E‑Learning limitiert ist. Rollenspiele anhand von standardisierten Patienten stellen eine wichtige Möglichkeit dar [50]. Rollenspiele affektiver Störungen, welche besonders hohe Anforderungen an die Vermittlung affektiver ToM stellen, werden von Experten als weniger realitätsnah beurteilt. Achtsamkeit konnte via Smartphone-App signifikant gebessert werden – passend zur besseren Vermittelbarkeit der kognitiven ToM [51].

Ein konstruktiver Umgang mit Empathie muss geübt werden – um negative direkte und indirekte Folgen abzuwenden [2, 8, 21, 30–32]: Mentorship-Programme, Fairness in der Verteilung der Arbeitslast, Anerkennung der Leistung und Rückhalt im Team, Maßnahmen, welche die Resilienz positiv beeinflussen und Möglichkeiten für Training und Weiterentwicklung wirken entgegen.

## Fazit für die Praxis

- Empathische Therapeuten lassen diese Fähigkeit in ihr therapeutisches Handeln einfließen und zeigen Wirkeffizienz.
- Prosoziales Verhalten wird bereits im Kindesalter in wiederholten Interaktionen mit den wichtigen Bezugspersonen vermittelt und ist an die Ausbildung einer sozialen Identität gekoppelt. Die Vermittlung von prosozialen und proempathischen klinischen Skills ist evidenzbasiert und effektiv, selbst wenn sie zu einem späteren Zeitpunkt im Leben erfolgt.
- Ein Empathietraining ist effektiv, wenn es auf die Vermittlung von Fähigkeiten wie Containment, Mentalisierungsfähigkeit und Emotionsregulation fokussiert, und darauf, eigene Erfahrungswelten nicht auf das Gegenüber zu übertragen (vgl. Abstinenz, Übertragung/Gegenübertragung).
